# Taches blanches choroïdiennes et sarcoïdose

**DOI:** 10.11604/pamj.2014.17.101.3566

**Published:** 2014-02-08

**Authors:** Hakima Elouarradi, Tachfouti Samira

**Affiliations:** 1 Université Mohammed V Souissi, Service d'Ophtalmologie A de l'hôpital des spécialités, Centre hospitalier universitaire, Rabat, Maroc

**Keywords:** Sarcoïdose, fond d′œil, taches blanches, sarcoidosis, fundus, white spots

## Image en medicine

La sarcoïdose est une affection multi-systémique caractérisée par un granulome épithélioïde sans nécrose caséeuse. Elle peut atteindre le système lymphatique médiastinal, les poumons, la peau, l'oeil et parfois le système nerveux central. L'atteinte oculaire du segment postérieur sous la forme de granulomes choroïdiens est très rare et ne représente que 5 % des cas d'atteintes ophtalmologiques. Nous rapportons le cas d'une femme de 54 ans suivie en médecine interne pour sarcoïdose qui s'est présentée aux urgences ophtalmologiques pour des épisodes transitoires de rougeur et de baisse de l'acuité visuelle de l'oeil droit depuis 2 semaines. L'acuité visuelle était à 3/10 et P8 au niveau de l'oeil droit et à 10/10 et P2 au niveau de l'oeil gauche. L'examen du segment antérieur des 2 yeux était strictement normal. L'examen du fond d'oeil mettait en évidence à droite une discrète hyalite et de nombreuses taches blanc jaunâtres choroïdiennes de petites tailles diffuses s'étendant en moyenne périphérie avec un aspect d'oedème maculaire (A). L'oeil gauche était normal. Cette observation illustre cette atteinte rare du segment postérieur au cours de la sarcoïdose dont il faut savoir évoquer le diagnostic en présence de telles lésions choroïdiennes désignées sous le nom de choroïdite multifocale. Une angiographie rétinienne à la fluorescéine réalisée objectivant et caractérisant l'atteinte choroïdienne et l'oedème maculaire (B) encore confirmée par la tomographie en cohérence optique (OCT) (C). Après un bilan exhaustif d'uvéite, Patiente a bénéficié d'injections sous conjonctivales de corticoïdes vu l'unilatéralité de l'atteinte, avec une nette amélioration de l'acuité visuelle.

**Figure 1:**
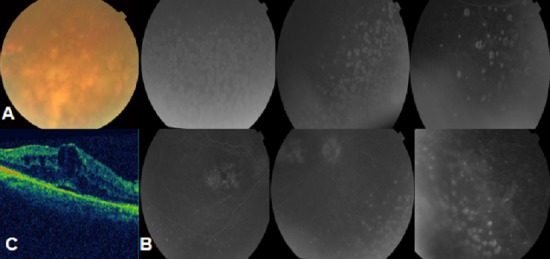
A) aspect du fond d’œil de l'œil droit : hyalite et taches blanc jaunâtres choroïdiennes; B) aspect angiographique de l'œil droit : choroïdite multifocale et œdème maculaire; C) aspect à la tomographie en cohérence optique: œdème maculaire cystoïde

